# Do-it-yourself: construction of a custom cDNA macroarray platform with high sensitivity and linear range

**DOI:** 10.1186/1472-6750-11-97

**Published:** 2011-10-25

**Authors:** Tom Boonefaes, Erica Houthuys, Rafael Van den Bergh, Seppe Vander Beken, Geert Raes, Peter Brouckaert, Patrick De Baetselier, Johan Grooten

**Affiliations:** 1 Department of Biomedical Molecular Biology, Ghent University, Sint-Pietersnieuwstraat 25, B-9000 Ghent, Belgium; 2 Department for Molecular Biomedical Research, VIB, Technologiepark 927, B-9052 Ghent, Belgium; 3 Department of Molecular and Cellular Interactions, VIB - Vrije Universiteit Brussel, Pleinlaan 2, B-1050 Brussels, Belgium; 4 Laboratory of Cellular and Molecular Immunology, Vrije Universiteit Brussel, Pleinlaan 2, B-1050 Brussels, Belgium; 5 Department of Immunology, Max Planck Institute for Infection Biology, Charitéplatz 1, D-10117 Berlin, Germany; 6 Department of Dermatology and Allergic Diseases, Ulm University Hospital, Maienweg 12, D-89081Ulm, Germany

## Abstract

**Background:**

Research involving gene expression profiling and clinical applications, such as diagnostics and prognostics, often require a DNA array platform that is flexibly customisable and cost-effective, but at the same time is highly sensitive and capable of accurately and reproducibly quantifying the transcriptional expression of a vast number of genes over the whole transcriptome dynamic range using low amounts of RNA sample. Hereto, a set of easy-to-implement practical optimisations to the design of cDNA-based nylon macroarrays as well as sample ^33^P-labeling, hybridisation protocols and phosphor screen image processing were analysed for macroarray performance.

**Results:**

The here proposed custom macroarray platform had an absolute sensitivity as low as 50,000 transcripts and a linear range of over 5 log-orders. Its quality of identifying differentially expressed genes was at least comparable to commercially available microchips. Interestingly, the quantitative accuracy was found to correlate significantly with corresponding reversed transcriptase - quantitative PCR values, the gold standard gene expression measure (Pearson's correlation test *p *< 0.0001). Furthermore, the assay has low cost and input RNA requirements (0.5 μg and less) and has a sound reproducibility.

**Conclusions:**

Results presented here, demonstrate for the first time that self-made cDNA-based nylon macroarrays can produce highly reliable gene expression data with high sensitivity and covering the entire mammalian dynamic range of mRNA abundances. Starting off from minimal amounts of unamplified total RNA per sample, a reasonable amount of samples can be assayed simultaneously for the quantitative expression of hundreds of genes in an easily customisable and cost-effective manner.

## Background

DNA arrays are widely used for the comprehensive gene expression analysis of an organism or sample. Arrays are available as high-density microarrays capable of covering the whole genome of an organism, and as low-density custom arrays containing a specific set of genes. High-density microarrays are mainly used during the stages of experimental discovery and hypothesis generation, custom arrays are suitable for hypothesis-driven research. Custom arrays allow researchers to focus on broad sets of genes or gene polymorphisms specific to particular cell populations, signalling pathways or disease conditions, while providing ultimate control over the experimental design. In general, specifically tailored macroarrays are more suitable than microarrays for diagnosis, drug discovery and validation, and for prognostic assessment of clinical treatments due to their low levels of background noise, flexibility, and lower price [1]. In contrast to gene expression analysis by reverse-transcriptase quantitative PCR (RT-qPCR), which is well-suited for sensitive analysis of a limited number of target genes, focused arrays allow cost-effective assessment of hundreds of targets in a large number of patients [2].

Various versions of DNA array methods exist with respect to probe substrate and detection method [3]. At present, commercially available custom DNA arrays are almost always printed on glass slides, and detection and quantification rely on measurement of fluorescence intensity of hybridised fluorochrome-labelled samples, with good to excellent sensitivity and reproducibility [4-6]. However, commercially available glass arrays are still not commonly used within the wider research community, as the need for highly specialised equipment restricts the application of this technology to a small number of dedicated laboratories. Additionally, the costs are still very high for large-scale studies. Moreover, most commercially available arrays still require amplification of the mRNA mixture before labelling and hybridisation [7]. Although linear amplification protocols appear to work reasonably well [8], the risk of skewing relative abundances when amplifying such a complex mixture remains a concern [9-11].

Nylon arrays are appreciated as a relatively economical and user-friendly alternative to other high-throughput gene expression technologies and have a high do-it-yourself potential [9]. To date, however, the features of commercial arrays have not been rivalled by in-house nylon arrays and little to no information is provided in the literature on custom assay parameters, such as detection limit, linear range, accuracy and reproducibility, or on practical issues concerning sample amount, hybridisation protocol and array construction [12,13]. The last detailed paper on this subject dates back to 1999 [3].

Here, we describe the construction of a customisable cDNA-based macroarray platform capable of expression profiling of sub-microgram amounts of unamplified total RNA. Included are a set of easy-to-implement optimisations leading to a substantial increase in sensitivity and linear range. Our custom system is quick, flexible and cost-effective. It is based on commercially available equipment and can be easily implemented in any conventional research laboratory.

## Results

### Limitations imposed by intrinsic material properties

**Figure 1:**
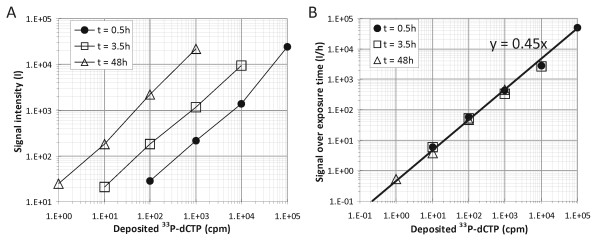
**Signal quantification of manually spotted volumes of ^33^P-dCTP**. A) Signal intensity of scanned spots after exposure for 30 min (full circles), 3 h 30 min (open squares) and 48 h (open triangles), as quantified by our analysis software. B) Signal intensity of the same scans expressed per hour of exposure.

Out of the macroarray systems, the least demanding setup in terms of laboratory infrastructure is the cDNA-based nylon membrane using ^33^P radioactivity for detection. cDNA probes are easy to generate by PCR, while printing on nylon membranes is more straightforward and requires less specific equipment than, for example, on glass chips. At the same time, use of ^33^P for labelling (^33^P-dCTP incorporated during reverse transcription) and a phosphor screen for detection should ensure high sensitivity and a wide dynamic range. To assess the limitations imposed by this setup on sensitivity, different amounts of ^33^P-dCTP were manually spotted on a nylon membrane, which was then exposed to a Bio-Rad phosphor screen and scanned in a conventional phosphor-imager at 50-μm resolution. Scans revealed that quantities down to ~1 cpm could be detected (Figure 1A). This corresponds to roughly 125,000 ^33^P-dCTP molecules or half a million total nucleotides, which indicates that as few as 1,000 hybridised copies of a 500-nt sequence can be detected. Additionally, when exposure time was varied, the intensity of the detected signal was found to be linear over time at a rate of 0.45 per hour per deposited cpm (Figure 1B). Therefore, the measured signal per hour of exposure corresponds directly to the amount of nucleotides on the membrane. As an implication hereby, Figure 1 illustrates that the linear range of detection will expand from about three to five log-values when all individual spots could be exposed for an optimal timeframe. A way to practically engage this option is to print cDNA-probes that generally generate high intensity spots on a separate membrane from the low intensity spots. This and other considerations for getting the most out of our custom cDNA-based nylon macroarray platform with ^33^P radioactivity are given below.

### Optimising the array design for image quantification and normalisation

After selection of the macroarray setup, we determined the optimal printing parameters and layout for our platform with regard to output data quality. Printing was performed using a Flexsys robotic workstation equipped with a 384-pin head. For six arbitrarily chosen genes, saturation of the radioactive signal of a single hybridisation reaction was reached for 10 ng of printed probe cDNA (not shown). To ensure maximal signals for each array, even in the case of small variations in the printing efficiency, we therefore chose to print 20 ng cDNA per spot, which corresponds to five transfers of the pin head.

**Figure 2:**
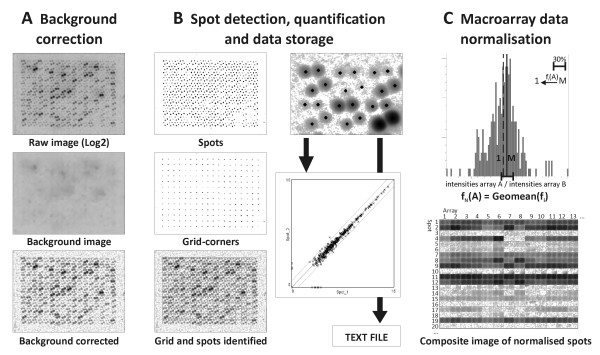
**Procedure flowchart for software image analysis**. A) Background correction, B) spot detection, quantification and data storage, and C) batch macroarray normalisation, including a composite picture of the normalised spots over all arrays in the batch. In each distribution histogram of spot intensity ratios between two arrays, the 30% genes with maximal area under the curve that thus represent the bulk gene-population with the lowest differential expression, were selected and the median thereof calculated. The normalisation factor for a given array pair was then defined by the difference of this median to 1, and the geometrical mean of all the pair-wise normalisation factors as the global normalisation factor for a given array. M = median, f_i_(A) = normalisation factor for A to correct against a given other macroarray, f_N_(A) = global batch normalisation factor for macroarray A.

The output of the phosphor-imager is a 16-bit image, which implies a range of 65,536 greyscales. Considering a background value of six for an unexposed screen, the resulting dynamic range of a single scan is four log-orders. To optimally exploit this dynamic range, a sensitive background correction is essential. In typical microarray experiments, quantified spot intensities are corrected with a local background value based on the intensities of pixels just outside the spot area. However, an intrinsic characteristic of radioactive signals is that spots are not well delineated. Consequently, local background values determined in the spot vicinity would be proportional to the actual spot intensity, which would lead to disproportionate overcorrection. We therefore constructed a background image by extrapolating the local minima across the entire array. This background image was then subtracted from the original image, which enabled us to visualise and reliably quantify spots that were marginally above the background value and in that way fully utilise the dynamic range (Figure 2A). Following background correction, the spots are automatically localised and quantified, and the data are exported to a text file by custom-made ImageJ add-on scripts (available upon request with the corresponding author) (Figure 2B). Also a scatter plot of the duplicate spots is generated for every analysed macroarray scan as an immediate indication of the overall quality (Figure 2B). In addition, a batch script features macroarray normalisation and generation of a series of log-transformed and normalised images of each spot over all the arrays analysed automatically (Figure 2C). Hereto, a custom normalisation approach was developed.

In commercial array platforms, normalisation is typically based on the assumption that the average expression ratio of either a subset or of all genes on the array should be equal to one. For whole-genome platforms this is a fair assumption, as the large background of non-differential genes compensates for the limited number of highly differential genes which may be present. However, highly differential genes might bias such normalisation when using focused arrays that are strongly enriched in genes that might be differentially expressed. Normalisation of our custom arrays was therefore performed by calculating an individual normalisation factor for each pair-wise combination of arrays, using the 30% least differentially expressed genes in order to minimise the likelihood of a normalisation bias (Figure 2C). The global normalisation factor for a given array in the batch was then calculated as the geometrical mean of all its individual pair-wise normalisation factors with the other arrays in the batch (Figure 2C). This approach is thus robust as long as not more than 70% of all genes on the array are differentially expressed between all samples in the batch, which is rarely the case. It is ideally suited for low density arrays as it does not require a set of housekeeping genes, the expression of which is assumed to stay stable within the experiment.

**Figure 3:**
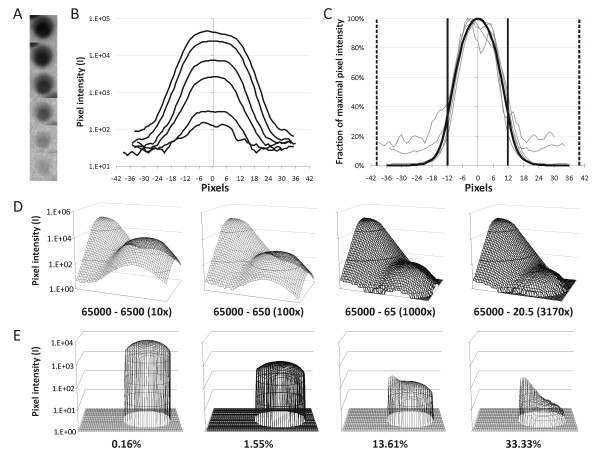
**Modelling of spot shape and intensity**. A) Log-transformed pictures of a selection of six macroarray spots of different intensities. B) Determination of the spot intensity profile from spots depicted in A by measurement of the pixel intensity as a function of the pixel distance from the spot centre. C) Determination of the general spot shape (black curve) by expressing the intensity at each distance for every spot as a fraction of the maximum intensity (grey lines). Full vertical lines represent 85% of the area under the curve at a diameter of 24 pixels (1.2 mm); dashed vertical lines represent the spot distance on the macroarray membrane (at 40 pixels = 2 mm). D) Mathematical modelling of the potential signal overspill between two adjacent spots 2 mm apart. Maximal intensity of neighbouring spot and measured spot are indicated, with the ratio between brackets. E) Signal measured within the 1.2-mm diameter around the lower intensity spot centre for the respective situations illustrated in panel D. Percentages denote which fraction of the integrated signal intensity is caused by overspill from the neighbouring maximal intensity spot.

As mentioned above, poor delineation of the spots is an intrinsic property of radioactive signals. This means that despite solid background correction during image analysis, spots should readily be printed at sufficient distances to ensure minimal overspill of signal between neighbouring spots, but close enough to maximise utilisation of the membrane area. To this end, spots with varying intensities were quantified (Figure 3A). Despite differences in spot intensity, the shape of the curve of pixel intensities throughout the spot diameter was invariant (Figure 3B). Modelling of this shape revealed that logarithmic decay of the signal started at 600 μm (12 pixels) from the spot centre, and that quantification of the spot area within a circle with 600 μm (12 pixel) radius yielded 85% of the total signal (Figure 3C). The minimal distance between two individual spots should therefore be at least 1.2 mm (2 × 600 μm). However, to limit signal overspill between neighbouring spots, we designed a medium-density layout with a minimal spot distance of 2 mm.

Subsequently, the potential overspill between spots at 2 mm distance was modelled (Figure 3D), based on the per pixel signal range between about 20 (background) and 65,000 (saturation) on a 16-bit tiff image and on the above modelled paraboloid spot shape (see also Figure 3C). With a tenfold difference in signal, i.e. with one spot reaching intensity saturation in its centre peak pixel and the second spot with a maximal intensity value of 6,500, 0.16% of the lower signal was caused by overspill from the adjacent spot with the higher signal (Figure 3E). This overspill value increased to 1.55% for a hundredfold difference between the maximal intensities of the adjacent spots and to 13.61% for a thousand fold difference (Figure 3E). To avoid a problematical 33.3% signal overspill, maximum intensities of neighbouring spots should not differ by more than about 3,000-fold (Figure 3E).

This restriction imposed by the current array design constrains the upper detection limit and therefore also imposes a limitation on the linear range of detection: highly abundant genes can lead to rapid saturation of the signal and to significant signal overspill when differing by over 3,000-fold in intensity from the neighbouring spot. To increase the linear range of detection, we separated typically high from typically low intensity probes over two membranes. These two sets were identified by hybridisations of pooled samples derived from a broad spectrum of macrophage activation models. In this way, high and low intensity membranes could be exposed for optimal durations without over-exposing the high intensity or under-exposing the low intensity spots. Additionally, large differences in signal intensity between neighbouring spots were precluded.

### Optimising the hybridisation conditions and use of sample RNA

Macroarray sensitivity depends significantly on both the quality and quantity of input ^33^P-dCTP labelled cDNA. From a research point of view, working with unamplified sub-microgram total RNA samples would be optimal. To evaluate this possibility, variations in the hybridisation and cDNA-synthesis protocols were evaluated.

We found that hybridisation concentrations could be scaled up from the traditionally used 10^6 ^cpm/ml to 10^7 ^cpm/ml without significant increase in background noise. This increase thus improved the detection sensitivity for transcripts of low abundance while maintaining absolute sensitivity. In a first optimisation assay, this tenfold increase in hybridisation concentration resulted in a tenfold increase of the specific signal (from 433 to 4,324) and a twofold increase (from 12 to 21) of the background noise intensity, thus implying a fivefold increase of the signal-to-noise ratio. In routine practice, this higher hybridisation concentration of 10^7 ^cpm/ml reproducibly generated average background intensity values of approximately 20. In practice, the high hybridisation concentration of up to 10^7 ^cpm/ml was reached with sub-microgram amounts (0.5 μg) of unamplified total RNA by hybridising the blots in standard conical 50-ml tubes with buffer volumes down to 2 ml.

**Figure 4:**
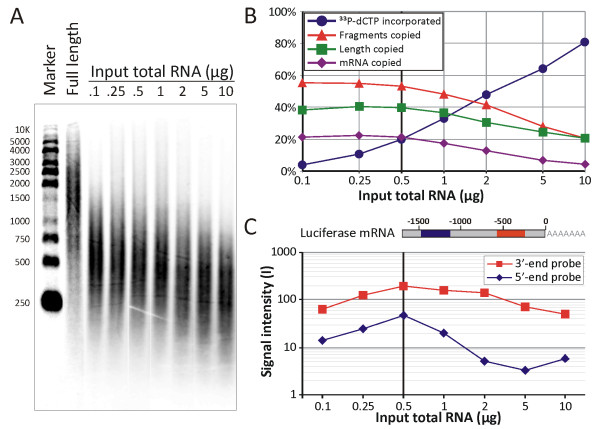
**Optimising reverse transcription for minimal sample input and maximal cDNA quality**. A) 10^5 ^cpm of cDNA samples from reverse transcriptase reactions with the indicated amounts of input total RNA were analyzed for length distribution by electrophoresis in 1% alkaline agarose. Full length synthesis was obtained by adding cold dCTP. B) Summary of the efficiency of cDNA synthesis and ^33^P-dCTP incorporation based on both liquid scintillation counting measured before and after the reverse transcriptase reaction and the cDNA length distribution, calculated from the alkaline gel picture in panel A as the weighted signal intensity per fragment length. The average fragment length, the ^33^P-dCTP incorporation values, as well as the number of fragments generated from different amounts of input total RNA were calculated and compared to the full-length synthesised sample. As a deduction thereof, also the mass proportion of mRNA in the sample that was transcribed was calculated, taking into account the assumptions made in the methods section 'Calculations, estimates and constants'. The black vertical line indicates the amount used in all other macroarray assays. Results shown in A-B are representative for two independent experiments. C) Membrane spot intensities (± variation on duplicate spots) generated by a given amount of luciferase control transcript spiked into different amounts of mouse peritoneal macrophage total RNA when hybridised at equal cpm with two different oligo-cDNA probes on the nylon membrane. Probes were designed to bind 3'-end or 5'-end sequences. The results confirm the quality of cDNA made from 0.5 μg total RNA (black vertical line) is suitable for array hybridisation.

At the level of cDNA synthesis, label incorporation and suitability for array hybridisation was analysed for different amounts of input RNA. Interestingly, analysis by alkaline gel electrophoresis (Figure 4A) revealed that cDNA generated from small amounts of total RNA, which contains less ^33^P-dCTP than higher amounts of total RNA (as was measured by liquid scintillation counting), contained more fragments of longer length (Figure 4B). Figure 4B shows that when 10 μg of total RNA were used, which is common for most macroarray platforms, only 21% of the fragments were copied to cDNA to an average 21% of fragment length (4% of mRNA copied), but when 0.5 μg of RNA was used, 53% of the fragments were copied to an average 40% of fragment length (21% of mRNA copied). Quite likely, small input amounts do not exhaust the reverse transcription reaction quickly, and so more and longer cDNA fragments are synthesised. It also implies that such cDNA should perform better in array hybridisations. To verify this, samples with different amounts of input RNA were spiked with equal amounts of control luciferase mRNA and subsequently labelled and hybridised at equal cpm amounts to membranes containing oligo-probes at the 3'-end and 5'-end of the luciferase transcript. Indeed, larger amounts of input RNA resulted in decreased signal intensity of the probe positioned more towards 5'-end, while the probe spots designed to recognise sequences located more towards the 3'-end showed more stable intensity (Figure 4C). This 'less-is-more' effect reached its optimum with 0.5 μg input RNA. When < 0.5 μg was used, spot intensities decreased equally for oligo-probes for 3'-end and 5'-end regions, probably because of simple dosage effects. The observation that less input RNA yields longer and better quality cDNA for array hybridisation is further supported by the performance of certain gene-probes on the macroarray (additional file 1: S1.TIF). Although the correlation was not absolute and most likely depended on each specific mRNA sequence (e.g. internal polyA-stretches), there was a clear correlation between the upstream position of the probe and the length-distribution of the cDNA. This also underscores that the observed effect is not specific to the luciferase control mRNA. In conclusion, increased assay sensitivity was achieved by (*i*) using 0.5 μg of total RNA to ensure effective label incorporation while yielding high quality cDNA with respect to length and composition, and (*ii*) using small hybridisation volumes (2 ml) in order to have high hybridisation concentrations.

### Assessment of efficiency and array sensitivity

The sensitivity of the macroarray platform was assessed on the basis of relative and absolute sensitivity. Relative sensitivity is the detection threshold for a specific mRNA within the total RNA pool whereas absolute sensitivity is the minimum number of molecules of a given mRNA that must be present in a sample of total RNA in order to produce a signal. Both relative and absolute sensitivity are a direct consequence of the labelling and hybridisation efficiencies. To measure relative and absolute sensitivity, samples were spiked with a serial dilution of control transcripts encoding either kanamycin or luciferase. As a measure of absolute sensitivity, spikes of luciferase mRNA between 54,700 and 170,000 transcripts (55 fg and 170 fg, respectively) could be reliably detected (Additional file 2: S2.PDF). Calculating from this absolute value the relative sensitivity requires an assumption of the amount of mRNA present within the total RNA sample and the number of mRNA transcripts contained herein. These values were calculated from the assay efficiency values, which yielded an approximate relative sensitivity in the range of 1/300,000 to 1/1,200,000 transcripts or - in most cases - the equivalence of one transcript per cell (Additional file 2: S2.PDF).

### Assessment of array reproducibility

**Figure 5:**
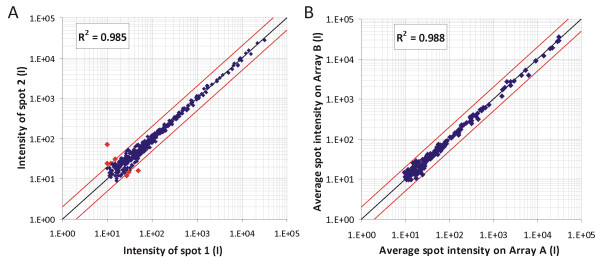
**Reproducibility of the macroarray platform**. Arrays were generated as described and hybridisations were performed with 33P-labelled samples derived from thioglycolate-elicited peritoneal excudate cells. Signal intensities of individual spots of duplicates on the same array (A), and average signal intensities of duplicate spots of two different arrays (B) were plotted against each other. Spots with an intensity deviating more than twofold in either direction are displayed in red.

All cDNA probes on the macroarray were spotted in duplicate, which made it possible to assess internal reproducibility. Plotting the duplicate spot intensities in a randomly selected hybridisation against each other showed that less than 3% of them deviated more than twofold from each other (Figure 5A). To assess the reproducibility between different arrays, technical repeats were performed by independently labelling a sample twice, followed by hybridisation to two separate arrays within the same batch of hybridisations (i.e. labelling reaction with the same batch of ^33^P-dCTP, identical exposure conditions). No genes showed more than a twofold deviation between the two arrays (Figure 5B).

### Validation of qualitative and quantitative array accuracy

To evaluate how the fully optimised custom array setup compares with other technologies, several analyses were performed in parallel on our mouse and human MAS macroarrays and on other well established gene expression platforms. Qualitative performance was assessed by comparing the ability of the human MAS focus array to pick up differentially expressed genes that were identified by the commercial CodeLink HWG platform. To this end, samples from monocytes of individual HIV-patients and healthy controls were hybridised in parallel to the MAS macroarray and to commercial CodeLink HWG microarrays, as described for a study published elsewhere [14]. Datasets composed of only genes represented on both arrays were filtered according to three criteria: (*i*) *p*-value as determined by Student's *t *test <0.05; (*ii*) a spot quality flag G ('good', a quality flag assigned by the CodeLink software package) in all CodeLink HWG arrays or variation between spot replicates ≤20% in all MAS arrays; (*iii*) a fold change between the means of the two groups ≥1.5. With this approach, both the CodeLink and MAS array platforms identified four genes as differentially expressed in the patient and control groups. The MAS array identified five other genes that were not significant according to the CodeLink analysis, whereas CodeLink microarrays did not identify any additional genes within the defined set of common genes (Table 1). All differentially expressed genes identified by both platforms as well as the additional genes picked up by our MAS array were confirmed to be true positives by an independent RT-qPCR analysis of the same samples. The qualitative identification of differentially expressed genes by our custom array platform is therefore at least comparable to commercially available setups.

**Table 1:** Differential gene-expression between monocytes from HIV-patients and healthy controls, compared to microarray and RT-qPCR results

Gene OGS	Gene Entrez ID	MAS-macroarray		CL-HWG		RT-qPCR
		*p*-value*	fold change	*p*-value*	fold change	fold change
CAPG	**822**	0.0375	-1.76	n.s.	1.09	-2.02
CCR1	**1230**	0.0240	2.16	n.s.	1.32	2.21
CDKN1A	**1026**	0.0012	1.91	0.0066	1.59	1.76
IL1F7	**27178**	0.0480	-1.58	n.s.	1.05	-2.12
NAMPT	**10135**	0.0381	2.34	0.0010	1.56	2.56
PDCD1LG2	**80380**	0.0087	-3.19	0.0065	1.68	-3.15
PTGER2	**5732**	0.0477	1.57	n.s.	1.22	1.85
STAT1	**6772**	0.0164	2.40	0.0004	1.52	2.25
YWHAZ	**7534**	0.0429	1.75	n.s.	1.06	1.51

OGS: Official Gene Symbol, MAS: Macrophage Activation State, CL-HWG: CodeLink Human Whole Genome microarray. * Student's t-test.

**Figure 6:**
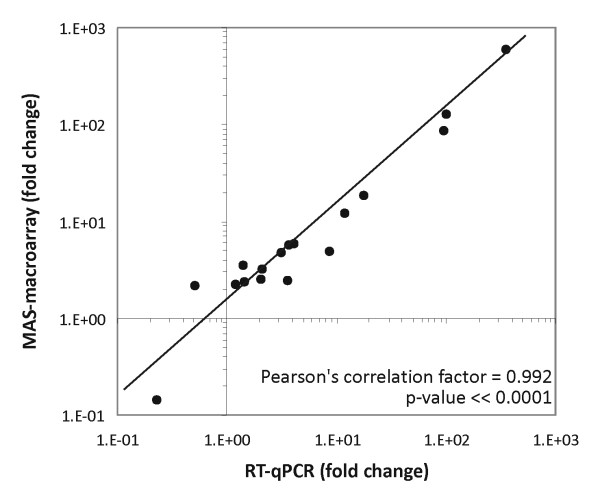
**Quantitative validation of the MAS array platform**. Fold changes as assessed by RT-qPCR and the MAS-macroarray platform for 17 individual genes differing at least twofold on the macroarray in a study of splenic macrophages from progressive tumour-bearing versus control mice. *p*-value of a Pearson correlation test is shown. MAS: Macrophage Activation State.

Finally, the quantitative performance of our platform was assessed by comparing MAS array and RT-qPCR expression data. Differential gene expression was analysed in a study of splenic macrophages from tumour-bearing *versus *control mice, as documented elsewhere [15]. Genes showing more than a twofold difference in expression in MAS macroarray profiling were analysed using RT-qPCR: fold changes were very similar between the two setups (*p *< 0.0001 according to a Pearson's correlation test), which shows the usefulness of the macroarray platform as a semi-quantitative gene expression analysis tool (Figure 6).

## Discussion

We developed a focused gene expression profiling platform capable of simultaneously assaying the qualitative and quantitative expression of hundreds of genes in a reasonably large amount of biological samples. Our system requires only basic laboratory equipment and can be used in common clinical and/or laboratory settings. All results discussed here are based on our Macrophage Activation State array (human and mouse) focusing on genes involved in the immunobiology of cells of macrophage lineage. However, the same setup can be readily applied to other species and/or other cell types or tissues.

In the scope of this paper, the macroarray setup and assay were optimised for the following crucial parameters:

### Linear range

A system designed for the simultaneous analysis of several hundreds of genes must be linear over an expression range that ideally covers the ~ 4.5 orders of magnitude of the mammalian transcriptome. Use of ^33^P-based labelling is the first factor in extending linear range; we show that the ^33^P signal is linear over the required range and that it remains linear over time. While radioactive labels ensure high sensitivity and a linear range, they suffer from the problem of overspill, which is not experienced with conventional fluorescent dyes: spots with high intensity can outshine and erroneously contribute to the intensity of neighbouring spots. To fully utilise the linear range inherent to the use of ^33^P, overspill must be minimised. To this end, spot quantification range and minimal spot distance were calculated and implemented. Using our setup, an erroneous 1.5-fold increase in signal intensity is theoretically possible only when the highest and lowest expressed genes are spotted adjacently.

A second challenge to our setup is that the inherent linear range of ^33^P is compromised by limits of the detection system. The phosphor-imager renders the exposed phosphor screens to a 16-bit tiff image, and saturation is therefore reached at an intensity of ~ 65,000. As background intensities are ~ 20 in this 16-bit image, the range is effectively reduced to ~ 3.5 orders of magnitude. This problem was solved by spotting genes of high and low abundance on different membranes, which are hybridised together but exposed separately for the optimal times. In this way, the phosphor-imager linear range of 3.5 orders of magnitude is required to cover only part of the transcriptome, and the signal per hour values of both membranes combined easily cover the 4.5 log-orders needed. Additionally, the problem of signal overspill is further reduced, because genes of high and low abundance are kept separate and will therefore not be printed adjacently.

### Sensitivity

Use of ^33^P-based labelling also allowed us to obtain high sensitivity. Furthermore, detection of low-abundant transcripts was enhanced, probably down to one out of a million, by increasing the hybridisation concentration to 10^7 ^cpm/ml, decreasing signal overspill, and generating more and longer cDNA from sub-microgram amounts of input RNA. Sensitivity was further enhanced by our image analysis approach to background correction, which is well-adapted for detection and quantification of radioactive signals marginally above background levels. Taken together, our setup can detect down to 50,000 fragments of a spiked mRNA species.

### Reproducibility

To avoid high signal variability due to differences in the quantity of printed probes, cDNA was spotted on the membranes at concentrations well above saturation levels. Additionally, we applied a 'less-is-more' approach to mRNA reverse transcription. Use of large amounts of input RNA in the reverse transcription/labelling reaction can quickly exhaust the reverse transcriptase reaction and result in very short cDNA fragments. This might render the sample prone to greater variability as a result of minute differences in reverse transcription efficiency, especially for genes for which the corresponding probe printed on the array is more distal from the 3'-end of the mRNA fragment. Use of smaller amounts of input RNA (0.5 μg) resulted in cDNA of higher quality in terms of fragment number and length.

### Cost-effectiveness

Using the proposed minimal spot distance of 2 mm, a 10 cm × 7 cm nylon membrane can accommodate 384 genes printed in duplicate, and multiple membranes can be used in the same hybridisation reaction. Expression of several hundred genes can therefore be analysed quantitatively in a sample of < 1 μg. Furthermore, the array platform described here can be constructed and used in most laboratories with standard equipment, which avoids the need to outsource valuable samples for transcriptome analysis and keeps costs low. Array printing could be the main bottleneck, as we have used a robotic work station to accurately spot each cDNA probe. However, the possibility of printing cDNA for microarray construction using conventional inkjet printing technology was first mentioned almost ten years ago [16]. The costs of the system presented here can be further reduced by stripping off the hybridised material and re-using the nylon membranes. We have determined empirically that stripping and reusing the membranes twice does not significantly affect their accuracy and reproducibility.

The array platform described here has many possible applications. First, it can be used downstream of genome-wide microarray analyses. The genes identified in a genome-wide gene expression analysis can be incorporated in a focused array, which can then be used for follow-up experiments or confirmation of gene expression results in a wider range of samples. Second, it can also serve as a rapid screening tool for complex phenotypes that cannot be characterised by using only one or even several parameters. Third, it can be used in clinical studies, which typically require many samples to achieve sufficient statistical power, as a substitute for costly microarray systems. In particular, our focused system could be a suitable alternative to many commercially available options when there is some knowledge of a collection of genes of interest or of a particular cell type under study.

## Conclusions

In conclusion, we have constructed a custom macroarray platform ideally suited for the investigation of an intermediate number of genes, in particular when sample material is scarce or when the study population is large. Our custom approach is robust and flexible. It has several advantages and fits well into present research laboratory practices.

## Methods

All technological optimisations were performed in an array setup focused on myeloid cell populations -the so-called Macrophage Activation State (MAS) macroarray - which was used in all optimisation experiments and quality assessment procedures described here.

### Array construction

For macroarray construction, a cDNA pool was generated by oligo-dT mediated reverse transcription of total RNA of monocytes/macrophages under various *in vitro *and *ex vivo *conditions. cDNA probes were generated by PCR amplification of the cDNA pool using gene-specific primers (Table 2). The PCR products were purified by filtration over Multiscreen PCR_96 _filter plates (Millipore, Billerica, MA, USA), resuspended in nuclease-free water, and separated by agarose gel electrophoresis to evaluate the size of the PCR products and efficiency of the PCR reaction. Products from failed PCR reactions (no PCR product or PCR product with more than one band or of an unexpected size) were excluded, and the remaining PCR products were air-dried overnight at 52°C and resuspended in 2× saline-sodium citrate (SSC) buffer with 0.4 M NaOH by five cycles of 30 s at 85°C and 30 s at 20°C. Macroarrays were prepared by spotting the PCR products in duplicate on 7 × 10 cm Hybond-XL membranes (Amersham GE Healthcare, Buckinghamshire, UK) using a Flexsys robotic workstation (Genomic Solutions, Ann Arbor, MI, USA) equipped with a 384-pin head, followed by cross-linking using UV light.

**Table 2:** Characteristics of gene-specific primers for probe cDNA generation

Parameter	Limits
**Primer length**	16-25 nt
**Primer Tm**	52-60°C
**Primer dTm**	≤ 2°C
**3' pentamer stability**	8.5 -kc/M
**Product size**	250-550 bp (tolerated 250-750 bp)
**Product location**	3' proximal 1000 nt (tolerated 1500 nt)
**Product Tm**	70-90°C

### ^33^P-dCTP labelling

For the sample labelling reaction, 0.125 μg oligo-dT T8 primer (Invitrogen, Carlsbad, CA, USA) was added to the total RNA sample, and the mixture was denatured for 10 min at 70°C. cDNA probes were then generated by reverse transcription with Superscript II reverse transcriptase (Invitrogen) in the presence of ^33^P-dCTP (0.05 mCi/sample; PerkinElmer, Waltham, MA, USA), dATP, dTTP and dGTP (each 100 μM; Invitrogen), and RNase inhibitor (Promega, MA, USA). After cDNA synthesis, 60 mM EDTA and 150 mM NaOH were added, the sample was incubated for 10 min at 42°C, after which 250 mM Tris-HCl (pH 6.8) was added. Subsequently, probes were purified using ProbeQuant G-50 Micro Columns (Amersham GE Healthcare) according to the manufacturer's instructions, and radioactivity incorporation was determined by liquid scintillation counting.

### Array hybridisation and data acquisition

Before hybridisation, macroarray membranes were washed in 2 × SSC and pre-incubated for 1 h at 42°C in 2 ml NorthernMax hybridisation buffer (Ambion, Austin, TX, USA) containing 40 μg/ml heat-denatured salmon testes DNA (Sigma-Aldrich, Saint-Louis, MO, USA). Probes were denatured for 5 min at 95°C. Hybridisation was performed at high probe concentration (10^7 ^cpm/ml) in 2 ml NorthernMax hybridisation buffer in 50 ml conical tubes for 20 h at 42°C with continuous rotation. Membranes were then washed three times in 2 × SSC buffer with 1% SDS, and twice in 0.6 × SSC with 1% SDS for 30 min at 68°C. Moistened filters were wrapped in plastic and exposed to a phosphor screen to reveal bound radioactivity. Phosphor screens were scanned with a phosphor-imager (BioRad Personal Molecular Imager FX, Hercules, CA, USA) at 50 μm resolution. Phosphor-imager output files were converted to 16-bit tiff images using the software application Quantity One (BioRad). The files were then for signal quantification using Java-based custom designed software on the freely available ImageJ platform (http://rsbweb.nih.gov/ij/).

### Samples

For optimisation and sensitivity testing of the custom array, total RNA was isolated from *ex vivo *LPS-stimulated mouse peritoneal macrophages with the Aurum total RNA mini kit (BioRad), according to manufacturer's instructions, and RNA yield was measured using a NanoDrop spectrophotometer (Thermo Scientific, Waltham, MA, USA). Where mentioned in the results section, samples were spiked with firefly luciferase or kanamycin control mRNA (Promega). Samples for the quality assessment of our array setup were collected and processed in the context of a study on monocyte-HIV interactions, as documented elsewhere [14], and in a study of splenic macrophages from BW-Sp3 tumour-bearing *versus *control AKR mice [15].

### cDNA alkaline gel electrophoresis

Samples of 10^5 ^cpm of the ^33^P-dCTP labelled cDNA were loaded in the slots of a 1% agarose gel made with running buffer (5 M NaOH/0.5 M EDTA) and run at 40 V for 3 h. The marker was generated by labelling of the phage λ HindIII marker with ^33^P-dCTP in a Klenow reaction, and the full length cDNA was generated by also adding 100 μM cold dCTP to the reverse transcriptase reaction. After neutralisation in 7% TCA, the gel was dried under vacuum, wrapped in foil, and exposed overnight to a phosphor screen. The resulting background-subtracted image was normalised for a constant total signal per lane for further analysis of fragment length distribution.

### Microarrays

Gene expression analysis of human samples was compared with datasets collected using CodeLink HWG bioarrays (Amersham Biosciences, Freiberg, Germany), which were used according to the manufacturer's instructions and were analysed as described elsewhere [14]. Datasets are available at the EMBL-EBI repository (accession number **E-MEXP-2255**).

### Quantitative RT-PCR

mRNA expression of genes of interest was examined using RT-qPCR. cDNA was prepared from 1 μg total RNA using oligo-dT and Superscript II reverse transcriptase (Invitrogen). Gene-specific primers different from the primers used for microarray probe generation were used in duplicate PCR reactions (Bio-Rad iQ SYBR Green Supermix) on a Bio-Rad MyCycler. Mouse gene expression was normalised against the housekeeping gene, ribosomal protein S12 (*Rps12*, Gene Entrez ID **20042**).

### Calculations, estimates and constants

• The lower prosphorscreen detection limit of the radioactive signal on a nylon Hybond-XL membrane, was empirically found to be 1 cpm or 6.23 × 10^-8 ^μl of a 10-μCi/μl batch of ^33^P-dCTP at 3 μCi/pmol, which therefore corresponds to 2.1 10^-19 ^moles, or about 125,000 molecules (when multiplied by Avogadro's constant).

• Starting from 0.5 μg total RNA, reverse transcription for radioactive labelling generated cDNA fragments with an average length of about 500 nucleotides as deduced from alkaline gel electrophoresis. This fragment length was used for further calculations.

• A 25% dCTP proportion in cDNA fragments is assumed.

• Where relevant, the proportion of mRNA in a sample of total RNA from mouse macrophages was estimated to be 5%.

## List of abbreviations used

MAS: Macrophage Activation State; RT-qPCR: Reverse Transcriptase - quantitative PCR

## Authors' contributions

TB carried out most macroarray design and assay optimisations, has written the image analysis scripts, and contributed to drafting this paper. EH participated in the qualitative and quantitative accuracy as well as reproducibility assessment of the platform and contributed to writing the manuscript. RVdB performed qualitative and quantitative assessment of the cDNA macroarrays and contributed to writing the manuscript. SVB participated in optimization of the reversed transcriptase procedure for macroarray analysis and in the sensitivity testing and contributed to writing the manuscript. GR, PB, PDB and JG have made substantial contributions to conception and design and have been involved in revising the manuscript critically. All authors read and approved the final manuscript.

## Supplementary Material

Additional file 1 **Figure S1 - cDNA quality depends on input RNA amount**. The left panel represents a scheme of the upstream distance on the mRNA transcript recognised by the gene-specific macroarray oligo-cDNA probe for a set of 23 genes. The right panel shows the log-transformed, normalised spots of this panel after hybridisation with the cDNA of different length distributions, generated from the indicated amounts of RNA input of a single sample. Although lower input RNA, resulting in longer cDNA, gives a good spot signal for all genes listed, this signal faints with higher input RNA, especially for spots with probes recognising more upstream transcript sequences. Some oligo-probes (e.g. for *Hnrpa2b1*, *Cd164*, *Vegfa *and *Cdkn1a*) seem to be more resistant to the effect of input RNA amount on the cDNA length distribution, which might be explained by the existence of internal polyA stretches or differences in secondary and tertiary mRNA folding. Click here for file

Additional file 2 **Macroarray efficiency and sensitivity calculations**. The pdf-file contains a figure (Figure S2) and a detailed description of the methodology as well as the used calculations to derive the macroarray efficiency parameters and both absolute and relative sensitivity parameters as they are summarized in the results section 'Assessment of efficiency and array sensitivity'. Click here for file
