# Supplementary material for: Do-it-yourself: construction of a custom cDNA macroarray platform with high sensitivity and linear range
Source: BMC Biotechnol. 2011 Oct 25;11:97. doi: 10.1186/1472-6750-11-97 (PMC3217856; doi:10.1186/1472-6750-11-97)
Supplement: Additional file 2 — Macroarray efficiency and sensitivity calculations. The pdf-file contains a figure (Figure S2) and a detailed description of the methodology as well as the used calculations to derive the macroarray efficiency parameters and both absolute and relative sensitivity parameters as they are summarized in the results section 'Assessment of efficiency and array sensitivity'. [file 1472-6750-11-97-S2.PDF]

Macroarray efficiency and sensitivity calculations

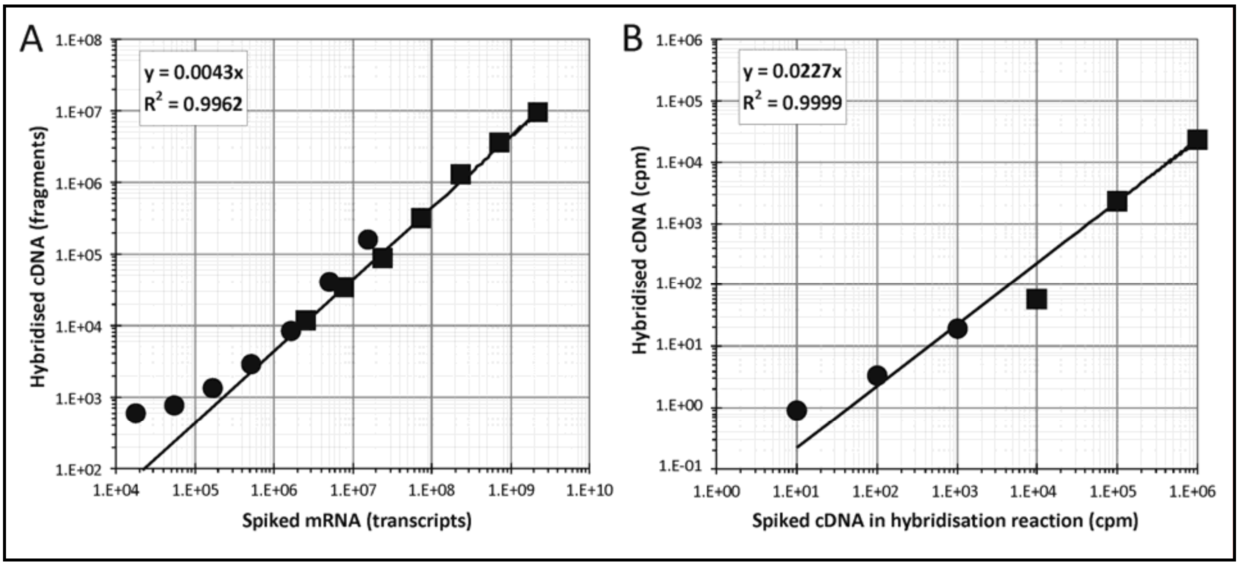

Figure S2 - Sensitivity and efficiency of the custom macroarray

A) A serial dilution of kanamycin (squares) and luciferase (circles) control mRNA was spiked into 0.5 µg total RNA samples. The spiked amount of transcripts is plotted against the amount of fragments (with a presumed 25% dCTP-content and average length of 500 nt) hybridised to the macroarray membrane. The amount of hybridised fragments was calculated from the measured spot intensities per hour as follows:

I /h * 0.45 = **X** cpm

= **X** * 125,000 ^{33}P-dCTP molecules

= (**X** * 125,000) / (0.25 * 500) fragments

Spikes down to less than 170,000 luciferase transcripts (= 170 fg) could be detected within the lower 1 cpm detection range of the macroarray nylon membrane. Therefore the **absolute sensitivity** is between this value and the lower spike of 54,700 transcripts (= 55 fg).

Above this lower detection limit, the yield of fragments after hybridisation is linearly dependent on the amount of control mRNA spiked into the sample. From the linear regression slope between the amount of spiked transcripts and that of measured fragments, **the macroarray efficiency** was calculated to be 4.3‰. This means that for every 1,000 control mRNA molecules spiked into the total RNA sample, 4.3 cDNA fragments (average 500 nt length) hybridised to the gene-specific oligo-cDNA probes on the array membrane.

B) The efficiency of the hybridisation reaction alone was assessed by adding serially diluted control kanamycin and luciferase cDNA spikes on a cpm basis to the hybridisation sample and plotting these cpm-spikes against the resulting signal (cpm = I /h * 0.45) after macroarray hybridisation. Again a linear correlation was observed and the regression constant revealed a 22.7‰ **hybridisation efficiency**. Thus for every 1,000 cpm of control cDNA added to the cDNA hybridisation sample, 22.7 cpm were selectively detected by the control transcript specific probes on the array membrane.

As a deduction, the **^{33}P-dCTP label incorporation** throughout the reverse-transcriptase reaction must have had an approximated efficiency of (4.3‰ / 22.7‰) = 19%, which is in the same range as the 21% that was empirically found in the manuscript under section: 'Optimising the hybridisation conditions and use of sample RNA' and is shown in Figure 4C.

Relative sensitivity

The relative sensitivity of the macroarray, defined as the lowest possible abundant transcript within the pool of mRNA molecules that can be detected, is directly dependent on the absolute sensitivity but also on the total amount of mRNA present in the sample. Assuming that the 0.5 µg of total RNA sample contains 5% mRNA, this is thus calculated as follows:

- **Absolute sensitivity of macroarray**
  = between 170 fg and 55 fg
- **mRNA content**
  = 5% of 0.5 µg
  = 25×10^6 fg mRNA
- **Estimated relative sensitivity of the macroarray**
  = between 170 fg / 25×10^6 fg and 55 fg / 25×10^6 fg
  = between 1 / 147,059 and 1 / 454,545

Thus transcripts with abundances up to about 1 out of 450,000 can be detected, assuming the mRNA content of the 0.5 µg sample equals 5% and that the transcript length approximates the average length in the mRNA pool. The mRNA content of the sample can also be calculated for these spiking experiments, as we measured the average cDNA-synthesis yield by liquid scintillation counting: $1.08 \times 10^7 \pm 0.14 \times 10^7$ cpm (n=10), and calculated the radioactive labeling efficiency to be 19% (see above):

$(1.08 \times 10^7 \pm 0.14 \times 10^7$ cpm) / 0,19 efficiency = $5.68 \times 10^7 \pm 0.74 \times 10^7$ cpm in case of 100% efficiency

$(5.68 \times 10^7 \pm 0.74 \times 10^7$ cpm) * (125,000 molecules ^{33}P-dCTP / cpm)

= $7.11 \times 10^{12} \pm 0.92 \times 10^{12}$ molecules ^{33}P-dCTP

= $(7.11 \times 10^{12} \pm 0.92 \times 10^{12})$ * 4 = $2.84 \times 10^{13} \pm 0.37 \times 10^{13}$ molecules dNTP

= $(2.84 \times 10^{13} \pm 0.37 \times 10^{13}$ molecules dNTP) / 500 nt average cDNA-length

= $5.68 \times 10^{10} \pm 0.74 \times 10^{10}$ cDNA fragments

= $5.68 \times 10^{10} \pm 0.74 \times 10^{10}$ mRNA template molecules within the sample

This calculated mRNA content allows for a more precise calculation of the relative sensitivity:

- **Absolute sensitivity of macroarray**
  = between 170,000 and 55,700 luciferase transcripts
- **mRNA content**
  = $5.68 \times 10^{10} \pm 0.74 \times 10^{10}$ transcripts
- **Calculated relative sensitivity of the macroarray**
  = between 170,000 / $(5.68 \times 10^{10} - 0.74 \times 10^{10})$ and 54,700 / $(5.68 \times 10^{10} + 0.74 \times 10^{10})$

  = between 1 / 291,022 and 1 / 1,173,867

So, the 5% mRNA content was probably an underestimation for the assayed macrophage cells, because the calculated relative sensitivity is higher (about 1 out of 300,000 to 1,200,000 transcripts) than the above (about 1 out of 150,000 to 450,000). As an additional appreciation of this value it is interesting to mention that, in our case, this meant that the macroarray was able to detect gene-expression of only one transcript per macrophage or less.
